# Supplementary material for: Implementation and Adherence of a Custom Mobile Application for Anonymous Bidirectional Communication Among Nearly 4000 Participants: Insights from the Longitudinal RisCoin Study
Source: Infect Dis Rep. 2025 Jul 24;17(4):88. doi: 10.3390/idr17040088 (PMC12385493; doi:10.3390/idr17040088)
Supplement: Supplementary file 1 [file idr-17-00088-s001.zip › idr-3684534-supplementary.pdf]

Contents

Supplement Table S1: Usage of RisCoin study app indicated by app active use among HCW (N=3816) and patients with IBD (N=163) 2

Supplement Table S2: Factors associated with being app non-users during the RisCoin study period (N=3816). 5

Supplement Figure S1: Flowchart of the RisCoin cohort based on app access and basic utilization of the short questionnaire in the app. 6

Supplement Figure S2: RisCoin app user journey – onboarding phase including download, activation, and login. 7

Supplement Figure S3: RisCoin app user journey – overview of the landing page and sections of patient record, research, and messages. 8

Supplement Figure S4: RisCoin app user journey – login via the desktop version of the app. The desktop version was available to all participants, the information and login details were provided on the second (back) page of the welcome letter. 9

Supplement Figure S5: RisCoin app user journey – overview of the results section including examples of the results presentation of anti-SARS-CoV-2 antibodies and neutralizing capacity against Omicron-BA.1 as viewed by the RisCoin participants. 10

References 11

Supplement Table S1: Usage of RisCoin study app indicated by app active use among HCW (N=3816) and patients with IBD (N=163)

	RisCoin sub-cohorts							
	HCW				Patient with IBD			
	Non-active users N=337 (9%)	Active users N=3479 (91%)	Total N=3816	P-value	Non-active users N=20 (12%)	Active users N=143 (88%)	Total N=163	P-value
**RisCoin HCW health status**				0.170[1]	N.a.	N.a.		
HCW plus[2]	148 (8%)	1664 (92%)	1812 (47%)		N.a.	N.a.		
HCW healthy[3]	189 (9%)	1815 (91%)	2004 (53%)		N.a.	N.a.		
**Gender**				**0.024[1]**				0.327[1]
Males	104 (11%)	879 (89%)	983 (26%)		9 (10%)	81 (90%)	90 (55%)	
Females	232 (8%)	2592 (92%)	2824 (74%)		11 (15%)	62 (85%)	73 (45%)	
**Age groups**				0.294[1]				0.321[1]
18-30	97 (9%)	1010 (91%)	1107 (29%)		3 (10%)	26 (90%)	29 (18%)	
31-40	89 (9%)	869 (91%)	958 (25%)		2 (5%)	36 (95%)	38 (23%)	
41-50	47 (7%)	642 (93%)	689 (18%)		6 (18%)	27 (82%)	33 (20%)	
51-60	77 (10%)	709 (90%)	786 (21%)		4 (10%)	35 (90%)	39 (24%)	
>60	27 (10%)	249 (90%)	276 (7%)		5 (21%)	19 (79%)	24 (15%)	
**BMI in 4 categories[4]**				0.187[1]				0.444[1]
Underweight	9 (8%)	101 (92%)	110 (3%)		0 (0%)	4 (100%)	4 (2%)	
Normal weight	229 (10%)	2167 (90%)	2396 (63%)		9 (11%)	71 (89%)	80 (49%)	
Pre-obesity	71 (8%)	845 (92%)	916 (24%)		6 (11%)	50 (89%)	56 (34%)	
Obesity all classes	27 (7%)	365 (93%)	392 (10%)		5 (22%)	18 (78%)	23 (14%)	
**Part-time employment**				**<0.001[1]**				0.182[1]
Yes	77 (7%)	1100 (93%)	1177 (31%)		2 (6%)	33 (94%)	35 (21%)	
No	259 (10%)	2376 (90%)	2635 (69%)		18 (14%)	110 (86%)	128 (79%)	
**Education**				**0.002[1]**				0.368[1]
Middle school diploma	30 (8%)	345 (92%)	375 (10%)		4 (12%)	29 (88%)	33 (21%)	
High school diploma	64 (7%)	848 (93%)	912 (24%)		3 (13%)	21 (88%)	24 (15%)	
Completed apprenticeship	52 (7%)	720 (93%)	772 (21%)		8 (20%)	32 (80%)	40 (25%)	
University degree	179 (11%)	1523 (89%)	1702 (45%)		5 (8%)	57 (92%)	62 (39%)	
**Healthcare occupation**				**<0.001[1]**				0.424[1]

	RisCoin sub-cohorts							
	HCW				Patient with IBD			
	Non-active users N=337 (9%)	Active users N=3479 (91%)	Total N=3816	P-value	Non-active users N=20 (12%)	Active users N=143 (88%)	Total N=163	P-value
Nurses	62 (7%)	838 (93%)	900 (24%)		0 (0%)	5 (100%)	5 (3%)	
Physicians	94 (14%)	582 (86%)	676 (18%)		0 (0%)	3 (100%)	3 (2%)	
Administration	52 (7%)	697 (93%)	749 (20%)		0 (0%)	2 (100%)	2 (1%)	
Others including TA, service staff	97 (8%)	1071 (92%)	1168 (31%)		2 (33%)	4 (67%)	6 (4%)	
Clinical scientists not working in patient care	27 (9%)	273 (91%)	300 (8%)		18 (12%)	129 (88%)	147 (90%)	
**Avoiding special foods i.e. due to allergy or intolerance**				**0.004**[1]				0.849[1]
Yes	9 (4%)	231 (96%)	240 (6%)		5 (13%)	33 (87%)	38 (23%)	
No	328 (9%)	3248 (91%)	3576 (94%)		15 (12%)	110 (88%)	125 (77%)	
**Participants with direct patient contact**				0.584[1]				0.600[1]
Yes	212 (9%)	2166 (91%)	2378 (62%)		1 (8%)	12 (92%)	13 (8%)	
No	120 (8%)	1309 (92%)	1429 (38%)		19 (13%)	131 (87%)	150 (92%)	
**COVID-19-related questions**								
**Contact with confirmed SARS-CoV-2 infected person ever**				0.169[1]				0.467[1]
Yes	278 (9%)	2875 (91%)	3153 (83%)		5 (16%)	26 (84%)	31 (19%)	
No or Unknown	51 (8%)	587 (92%)	638 (17%)		15 (11%)	117 (89%)	132 (81%)	
**Intensity of clinical symptoms after the 2nd COVID-19 vaccination**				0.502[1]				0.469[1]
None	145 (9%)	1447 (91%)	1592 (43%)		13 (13%)	86 (87%)	99 (63%)	
Mild or moderate complaints	151 (9%)	1606 (91%)	1757 (47%)		4 (7%)	50 (93%)	54 (34%)	
Severe complaints	26 (7%)	335 (93%)	361 (10%)		1 (20%)	4 (80%)	5 (3%)	
**Vaccinated against influenza during the last flu season**				0.390[1]				0.552[1]
Yes	161 (8%)	1802 (92%)	1963 (53%)		14 (14%)	84 (86%)	98 (64%)	
No	158 (9%)	1599 (91%)	1757 (47%)		6 (11%)	49 (89%)	55 (36%)	
**Pollen allergy**				0.087[1]				0.393[1]
Yes	87 (8%)	1072 (92%)	1159 (31%)		8 (16%)	43 (84%)	51 (32%)	
No	243 (9%)	2397 (91%)	2640 (69%)		12 (11%)	98 (89%)	110 (68%)	
**Smoking status (consumption of tobacco products e-cigarettes hookah pipe)**				0.764[1]				0.209[1]
Current smoker	55 (8%)	603 (92%)	658 (18%)		18 (69%)	8 (31%)	26 (19%)	

	RisCoin sub-cohorts							
	HCW				Patient with IBD			
	Non-active users N=337 (9%)	Active users N=3479 (91%)	Total N=3816	P-value	Non-active users N=20 (12%)	Active users N=143 (88%)	Total N=163	P-value
Non-smoker/Previous smoker	270 (9%)	2826 (91%)	3096 (82%)		63 (56%)	50 (44%)	113 (81%)	
**Self-perceived stress, PSQ>33**[5]				**0.031**[1]				0.490[1]
Yes	202 (10%)	1906 (90%)	2108 (56%)		12 (14%)	73 (86%)	85 (53%)	
No	128 (8%)	1558 (92%)	1686 (44%)		8 (11%)	68 (89%)	76 (47%)	

Results were presented in frequency (n) and row percentage (%) for the utilization frequency of RisCoin study app indicated by the number of entries through the short questionnaire: Active app users with ≥1 entries. Missing data, resulting from self-reporting, was evident as differences between the sums of subcategories and the total N.

1) P-values obtained by Pearson's Chi-square test to indicate significant differences in the proportion of non-active users (or active users) across different categories of a specific factor. P-values ≤ 0.05 were considered statistically significant.

2) HCW-plus includes all health care workers, who reported regular medication intake or at least one of the following underlying diseases: cardiovascular disease, chronic pulmonary disease, diabetes mellitus, thyroid dysfunction, hypothyroidism, chronic renal disease, renal insufficiency, chronic hepatic or gastrointestinal disease, chronic neurological disease or disorder, cancer, transplantation, chronic hematological disease, rheumatological disease, or primary immunodeficiency disorders.

3) HCW-healthy includes all health care workers, who did not report any underlying disease or any medication intake at enrolment.

4) BMI categories were obtained by applying the WHO criteria [1]

5) PSQ score: "Perceived Stress Questionnaire" Score; Participants filled out the 20-item validated version of the PSQ within the baseline questionnaire for RisCoin; we use the score of 33 as benchmark per the validation study of Fliege et al., where the score of 33 was determined for the healthy German population [2–4].

Abbreviations: HCW: health care workers, IBD: Inflammatory bowel disease, PSQ: Perceived stress questionnaire

Supplement Table S2: Factors associated with being app non-users during the RisCoin study period (N=3816).

Effect	OR	95% Confidence Limits		p-Value
**Age** (years)	1.010	1.000	1.020	**0.0443**
**Gender** (Males vs Females)	1.115	0.849	1.464	0.4332
**RisCoin HCW health status** (HCW plus [1] vs HCW healthy [2])	0.861	0.670	1.106	0.2411
**Healthcare occupation** (Physicians vs Administration)	2.839	1.909	4.222	**<.0001**
**Healthcare occupation** (Physicians vs Clinical scientists not working in patient care)	2.229	1.351	3.676	**0.0017**
**Healthcare occupation** (Physicians vs Others including TA, service staff etc.)	2.102	1.501	2.946	**<.0001**
**Healthcare occupation** (Physicians vs Nurses)	2.663	1.830	3.875	**<.0001**
**Contact with confirmed SARS-CoV-2 infected person** (No/Unknown vs Yes)	1.306	1.013	1.684	**0.0393**
**Vaccinated against influenza during the last flu season** (No vs Yes)	1.376	1.074	1.764	**0.0117**
**Self-perceived stress, PSQ score [3] >33** (Yes vs No)	1.332	1.045	1.698	**0.0208**

Odds ratios (OR) with 95% confidence intervals (95% CI) were obtained from the final multivariable logistic regression adjusted for gender, age, health status of health care workers with or without underlying disease (HCW-plus, HCW-healthy, respectively) to determine factors associated with being app non-users during the RisCoin study period. P-values were determined using the Wald Chi-Square test to assess the significance of the odds ratio (OR).

1) HCW-plus includes all health care workers, who reported regular medication intake or at least one of the following underlying diseases: cardiovascular disease, chronic pulmonary disease, diabetes mellitus, thyroid dysfunction, hypothyroidism, chronic renal disease, renal insufficiency, chronic hepatic or gastrointestinal disease, chronic neurological disease or disorder, cancer, transplantation, chronic hematological disease, rheumatological disease, or primary immunodeficiency disorders.

2) HCW-healthy includes all health care workers, who did not report any underlying disease or any medication intake at enrolment.

3) PSQ score: "Perceived Stress Questionnaire" Score; Participants filled out the 20-item validated version of the PSQ within the baseline questionnaire for RisCoin; we use the score of 33 as benchmark per the validation study of Fliege et al., where the score of 33 was determined for the healthy German population [2–4].

Abbreviations: HCW: health care workers, IBD: Inflammatory bowel disease, PSQ: Perceived stress questionnaire

Supplement Figure S1: Flowchart of the RisCoin cohort based on app access and basic utilization of the short questionnaire in the app.

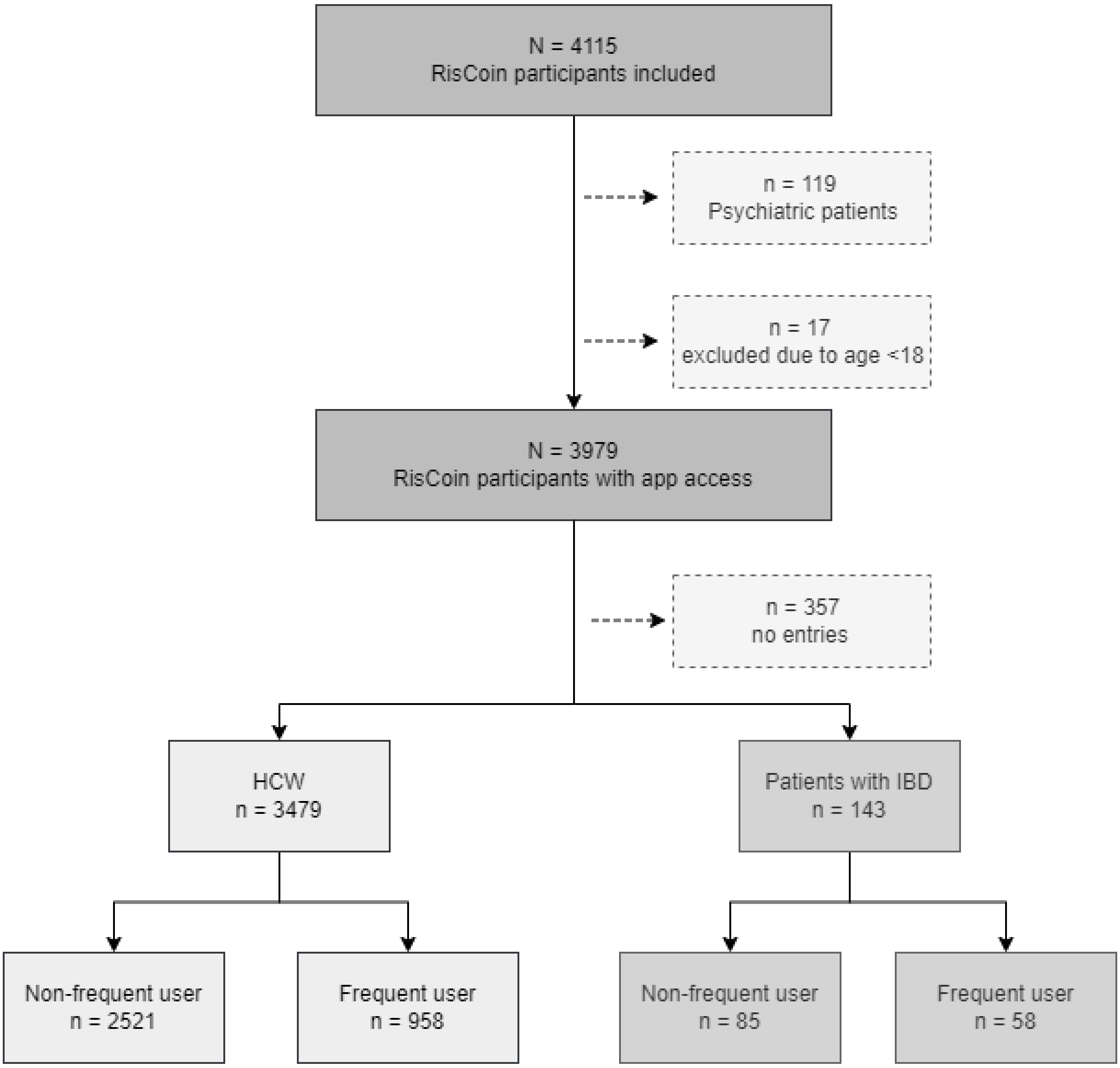

Supplement figure 1 presents the flowchart of the RisCoin cohort in terms of app access and basic utilization frequency. Following the exclusion of the psychiatric and underage patients, the analysis cohort consisted of 3979 RisCoin participants. Psychiatric patients were immunologically healthy patients with mental disorders from the Department of Psychiatry of the LMU University Hospital and were recruited as disease control group [2].

Supplement Figure S2: RisCoin app user journey – onboarding phase including download, activation, and login.

The app was available for download in the Apple and Play Store. Following the download, the landing page (below) provided overview of the three sections of the app – patient record, research and messages

Participants were then invited to activate the app using the code for one of the 5000 participant RisCoin profiles randomly assigned to them during onboarding. The code allowed for using the app without any entering personal information.

Scanning the QR code revealed the user data of the respective participant profile. This data presented the link between the app profile and the respective RisCoin participant profile in the database. This partial step did not require any action by participants.

Following the establishment of the connection between the app profile and the RisCoin database, participants were invited to create a password for the app and to select the option of biometric login via fingerprint.

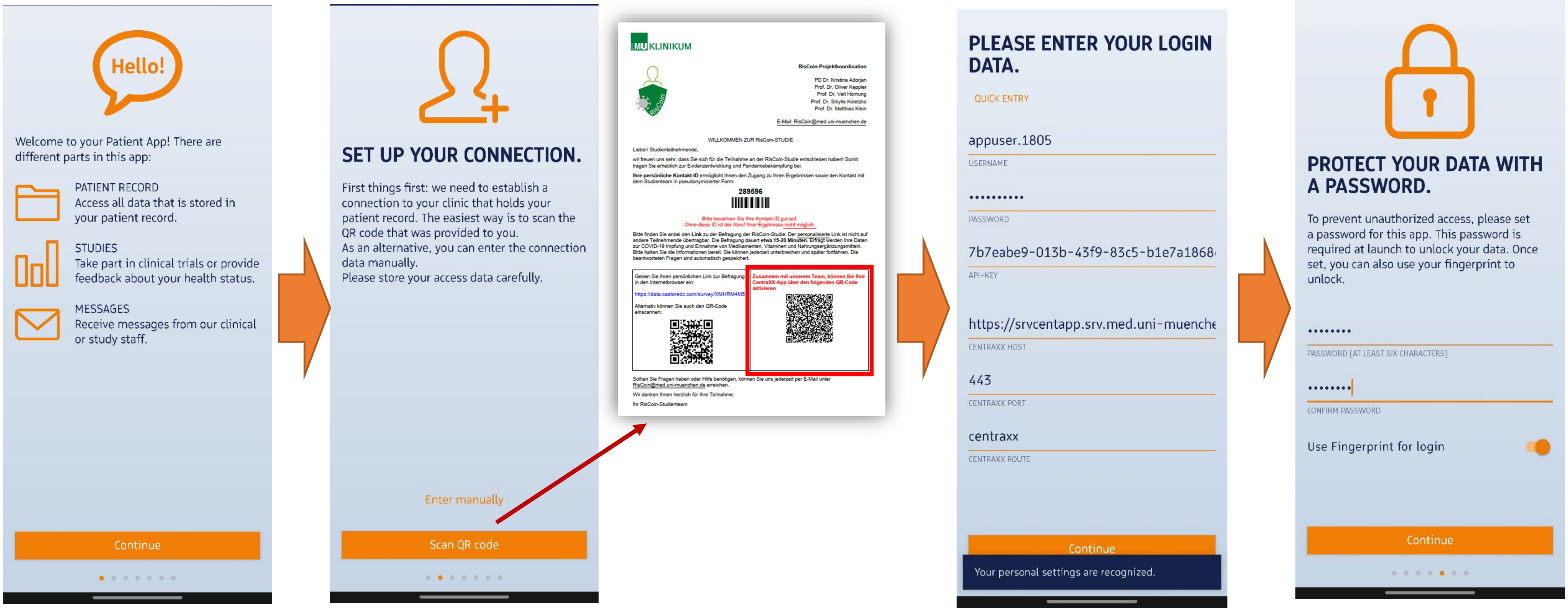

Supplement Figure S3: RisCoin app user journey – overview of the landing page and sections of patient record, research, and messages.

Landing page of the activated app following login with the password set by the participant or via fingerprint

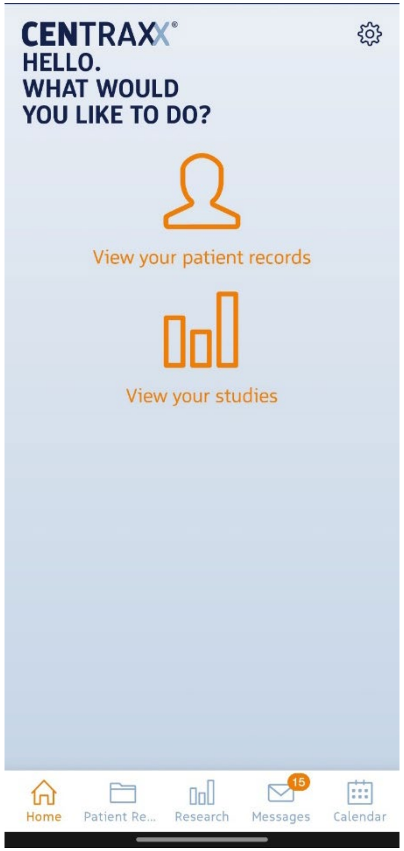

Sub menu of the section „Patient record“ containing master data, consents, episodes, diagnoses (unused), procedures, documents, measurements, samples, studies

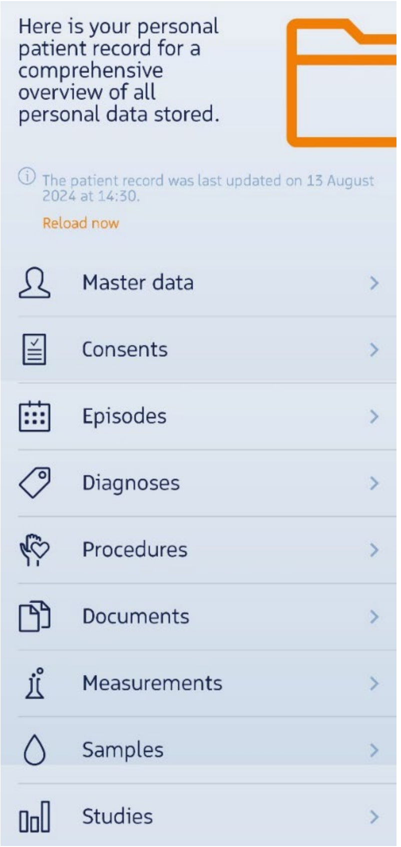

The section „Research“ included the weekly survey that allowed that study team to receive notifications of any changes in the participants‘ infection or vaccination status. Participants were able to view all weekly surveys previously submitted by them

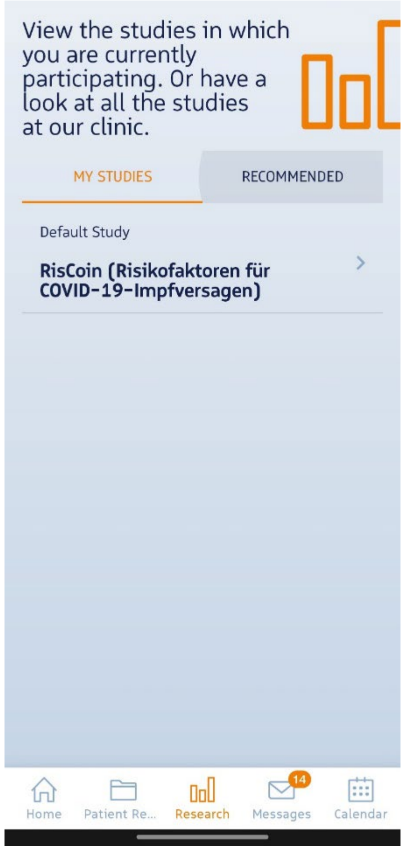

The section „Messages“ includes all messages sent by the study team as well as all bidirectional communication between the participant and the team. The participants were identified solely by their Contact-ID and no personal data were exchanged.

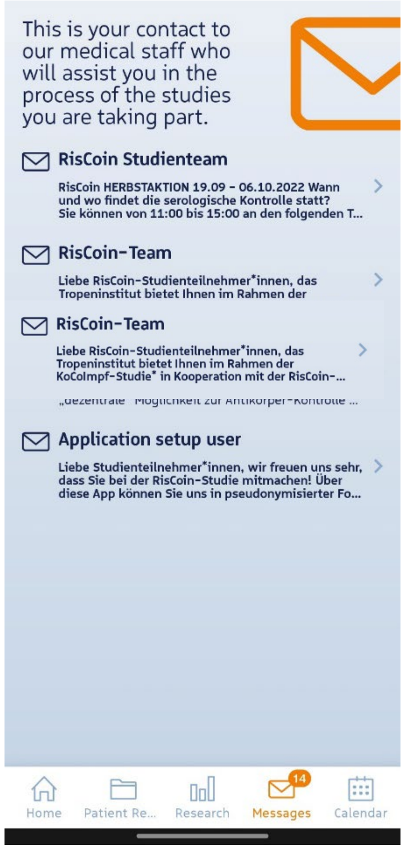

Supplement Figure S4: RisCoin app user journey – login via the desktop version of the app. The desktop version was available to all participants, the information and login details were provided on the second (back) page of the welcome letter.

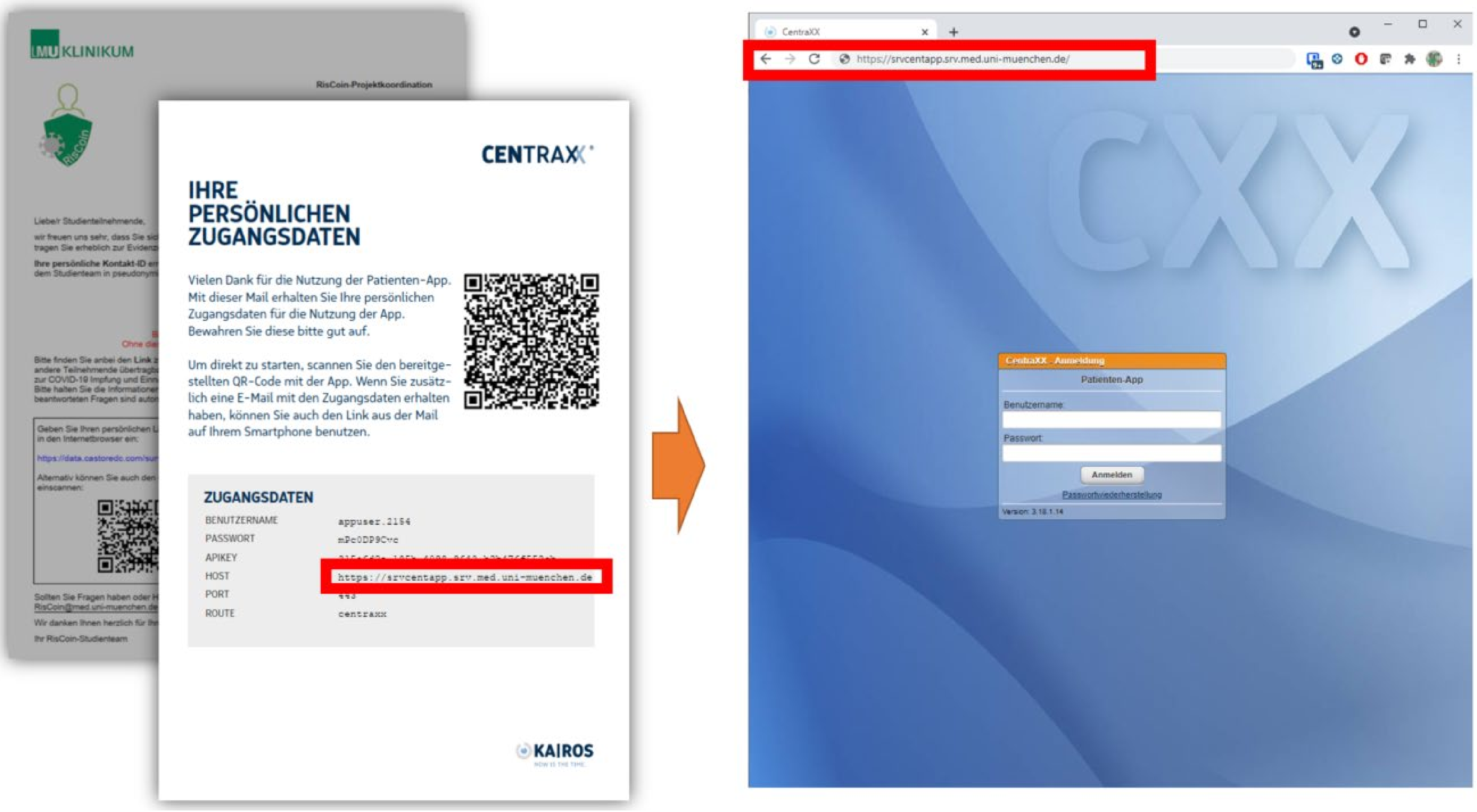

Supplement Figure S5: RisCoin app user journey – overview of the results section including examples of the results presentation of anti-SARS-CoV-2 antibodies and neutralizing capacity against Omicron-BA.1 as viewed by the RisCoin participants.

An overview of all results communicated to participants during the study (incl. Follow-ups)

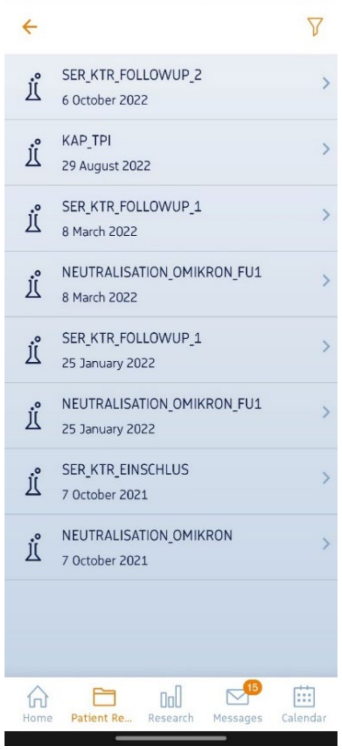

Result from baseline data collection: neutralizing capacity against Omicron-BA.1

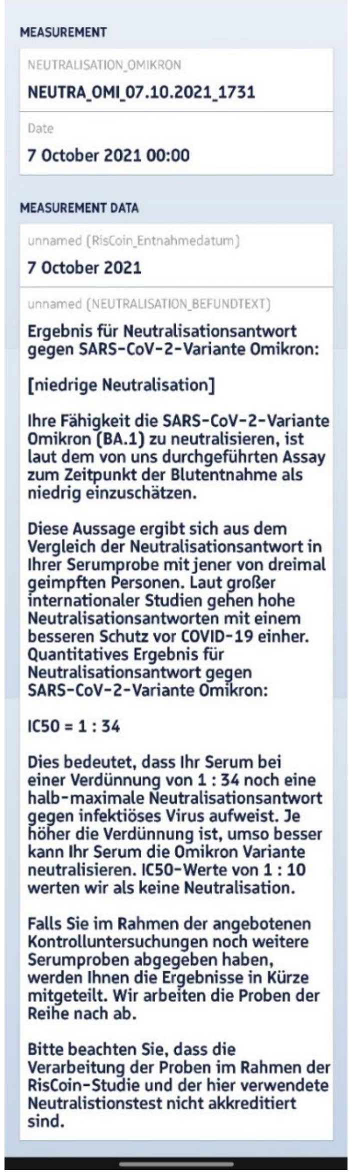

Result from baseline data collection: anti-SARS-CoV-2 antibodies

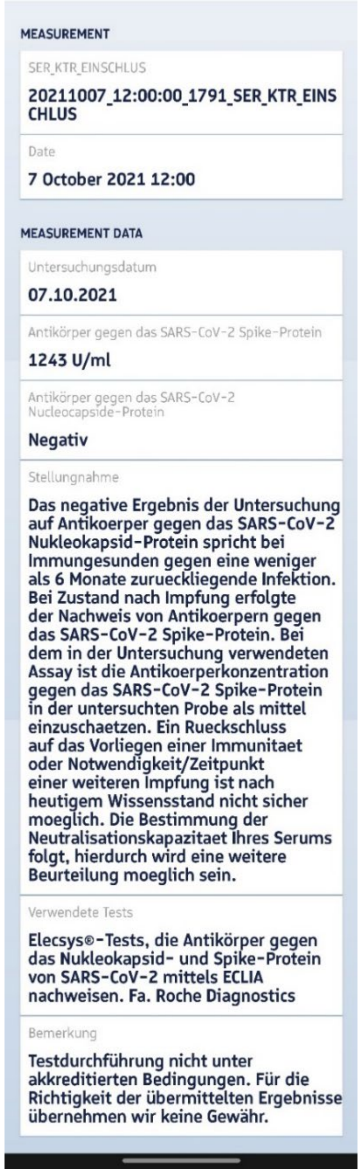

References

1. World Health Organization. *Obesity - Preventing and Managing the Global Epidemic: Report on a WHO Consultation*. Geneva: World Health Organization, 2000.
2. Koletzko S, Le Thi TG, Zhelyazkova A, et al. A prospective longitudinal cohort study on risk factors for COVID-19 vaccination failure (RisCoin): methods, procedures and characterization of the cohort. *Clin Exp Med* 2023.
3. Fliege H, Rose M, Arck P, et al. The Perceived Stress Questionnaire (PSQ) reconsidered: validation and reference values from different clinical and healthy adult samples. *Psychosom Med* 2005; 67: 78–88.
4. Fliege H, Rose M, Arck P, et al. Validierung des “Perceived Stress Questionnaire“ (PSQ) an einer deutschen Stichprobe. *Diagnostica* 2001; 47: 142–152.
